# Prevalence of Polycystic Ovary Syndrome Phenotypes and Their Association With Vitamin D Levels, Insulin Resistance, and Homeostasis Model Assessment of Insulin Resistance in Women With Polycystic Ovary Syndrome: A Cross-Sectional Analysis

**DOI:** 10.7759/cureus.95156

**Published:** 2025-10-22

**Authors:** Anupama Bahadur, Tuba Afreen, Ayush Heda, Rajlaxmi Mundhra, Manisha Naithani, Rashmi Verma, Madhavi Latha Ratala, Pragati Jakhar, Anushka Vishwakarma, Nilofer Khan

**Affiliations:** 1 Obstetrics and Gynaecology, All India Institute of Medical Sciences, Rishikesh, Rishikesh, IND; 2 Gynaecologic Oncology, All India Institute of Medical Sciences, Rishikesh, Rishikesh, IND; 3 Biochemistry, All India Institute of Medical Sciences, Rishikesh, Rishikesh, IND

**Keywords:** anti-müllerian hormone, hyperandrogenism, metabolic syndrome, obesity in pcos, pcos phenotypes

## Abstract

Objective: The objective of this study is to evaluate the association of polycystic ovarian syndrome (PCOS) phenotypes with various anthropometric, hormonal, and metabolic parameters and to explore the correlation between vitamin D deficiency and insulin resistance (IR) in women with PCOS.

Study design: This prospective cross-sectional study was conducted over 10 months, including 212 women diagnosed with PCOS based on the Rotterdam criteria. Participants were classified into four phenotypes, and comprehensive anthropometric measurements, hormonal assessments, and metabolic evaluations were performed. Spearman's rank correlation coefficient was used to assess the correlation between homeostasis model assessment of insulin resistance (HOMA-IR) and vitamin D levels (ng/mL). A p-value of less than 0.05 was considered statistically significant.

Results: Phenotype D (n=104, 49.06%) was the most common among participants, followed by Phenotypes A, C, and B. BMI and serum anti-Müllerian hormone (AMH) levels showed significant associations with PCOS phenotypes, with the highest BMI observed in Phenotype A; 53.8% (n=114) of participants had vitamin D deficiency. A significant negative correlation was found between vitamin D levels and IR (correlation coefficient: -0.207), as measured by the HOMA-IR, suggesting that lower vitamin D levels are associated with higher IR.

Conclusion: The study demonstrates significant associations between PCOS phenotypes and key anthropometric and hormonal parameters, particularly BMI and serum AMH levels. Additionally, vitamin D deficiency is prevalent in women with PCOS and is correlated with increased IR. These findings may contribute to the development of predictive models for metabolic abnormalities in PCOS and underscore the need for further research with larger, more diverse populations to better understand the complex pathogenesis of PCOS.

## Introduction

Polycystic ovarian syndrome (PCOS) is the most prevalent endocrine disorder, affecting approximately 5-10% of women of reproductive age [1]. This condition is multifactorial and genetically complex, with a challenging pathophysiology and an unclear etiology [2]. As the most common hormonal and metabolic disorder in women of childbearing age, PCOS is characterized by a variety of symptoms, including menstrual irregularities, clinical and biochemical signs of hyperandrogenism, and polycystic ovaries. The syndrome is driven by elevated ovarian and adrenal androgen secretion, resulting in hyperandrogenic symptoms such as hirsutism, acne, alopecia, and menstrual disturbances. Moreover, PCOS is often accompanied by significant metabolic disturbances, including hyperinsulinemia, insulin resistance (IR), and central obesity. Hyperandrogenism and hyperinsulinemia are key indicators of the hormonal imbalance that defines PCOS [3].

The Rotterdam criteria, widely recognized for diagnosing PCOS, outline four distinct phenotypes of the condition [4]. These phenotypes provide a framework for classifying the severity of PCOS: Phenotype A (Frank/Classic type) involves hyperandrogenism, oligo-ovulation/anovulation, and polycystic ovaries; Phenotype B (Non-polycystic type) is characterized by hyperandrogenism, oligo-ovulation/anovulation, but with normal ovaries; Phenotype C (Ovulatory type) includes hyperandrogenism and polycystic ovaries with regular menstrual cycles; and Phenotype D (Mild or normo-androgenic type) features oligo-ovulation/anovulation with normal androgen levels and polycystic ovaries. Each phenotype exhibits a unique metabolic and endocrinological profile, making it crucial to understand these variations to identify potential complications and optimize management strategies [5].

PCOS is frequently associated with a high prevalence of IR and obesity. It is estimated that around 40% of women with PCOS exhibit IR, with obesity further exacerbating the condition’s medical consequences [6]. Over time, this may lead to complications such as type 2 diabetes mellitus, metabolic syndrome, cardiovascular disorders, and obesity-related cancers, including endometrial cancer. Various indicators, such as homeostatic model assessment of insulin resistance (HOMA-IR), adiponectin levels, leptin, and other surrogate markers, have been evaluated to better understand IR in PCOS [7]. Additionally, vitamin D deficiency is commonly observed in PCOS patients and is associated with reproductive dysfunction, metabolic alterations, and mental health issues [8]. Serum vitamin D levels are inversely correlated with androgen levels, IR parameters, and body fat. Emerging research suggests that vitamin D insufficiency may contribute to the pathophysiology of IR and metabolic syndrome in PCOS through mechanisms involving the apoptotic pathway, growth and differentiation, and insulin metabolism [9].

This study aims to determine the prevalence of different PCOS phenotypes and to explore the correlations between vitamin D levels, IR, and HOMA-IR across these phenotypes.

## Materials and methods

Study design

This prospective cross-sectional investigation was conducted over a 10-month period from November 2020 to August 2021, following approval from the Institute Ethics Committee (AIIMS/IEC/20/772). The present study included women aged 18 to 38 years diagnosed with PCOS based on the Rotterdam criteria, who provided informed written consent for participation. Exclusion criteria included individuals with endocrine disorders such as diabetes mellitus and/or hyperprolactinemia, those with a history of using insulin-sensitizing agents within the past three months, and those with a history of hormonal drug use or ovarian surgery.

Clinical assessment

Women aged 18 to 38 presenting with PCOS symptoms at the Gynecology Outpatient Department (OPD) of our institution were evaluated for eligibility based on the Rotterdam criteria. Women aged 18-38 years were included to capture the reproductive age group most affected by PCOS and to minimize variability associated with perimenopausal or adolescent hormonal fluctuations [10]. Each participant underwent a comprehensive clinical examination, including the measurement of anthropometric parameters. These measurements included height (in centimeters), weight (in kilograms), hip circumference (measured at the widest part of the hips), and waist circumference (measured at the level of the umbilicus). Body mass index (BMI) was calculated from these measurements, with overweight classified using the Asian BMI range of 23.0-24.9 kg/m², and obesity defined as a BMI of ≥25 kg/m² [11]. Additionally, hirsutism was scored on a scale of 0-36, and the global acne score was recorded [12]. A detailed gynecological examination, including a per-vaginal bimanual examination, was conducted to rule out local pelvic pathology, except in adolescents and unmarried women.

Metabolic and hormonal analysis

Biochemical and hormonal assessments were performed during the follicular phase of the menstrual cycle (days 2-5). The analysis included fasting and postprandial blood glucose levels, fasting and postprandial serum insulin levels, and the calculation of HOMA-IR. Additionally, serum levels of follicle-stimulating hormone (FSH), luteinizing hormone (LH), testosterone, and vitamin D were measured using the chemiluminescence immunoassay (CLIA) method [13].

Statistical analysis

Data were analyzed using IBM SPSS Statistics for Windows, Version 21 (Released 2012; IBM Corp., Armonk, New York, United States). Categorical variables were presented as numbers and percentages (%), while quantitative data were reported as means ± standard deviation (SD) or as medians with interquartile range (IQR). Quantitative variables with non-normal distribution were analyzed using the Kruskal-Wallis test, while those with normal distribution were analyzed using ANOVA. Qualitative variables were analyzed using Fisher’s exact test. The Spearman rank correlation coefficient was used to assess the correlation between HOMA-IR and vitamin D levels (ng/mL). A p-value of less than 0.05 was considered statistically significant.

## Results

Study population

Over the 10-month study period, a total of 212 women diagnosed with PCOS based on the Rotterdam Criteria were enrolled. The mean age of the participants was 23.58 ± 4.6 years. The baseline characteristics of the study population are detailed in Table 1. The patients were further categorized into four distinct PCOS phenotypes. Phenotype D was the most prevalent, observed in 104 patients (49.06%), followed by Phenotype A in 73 patients (34.43%), Phenotype C in 18 patients (8.49%), and Phenotype B was the least common, found in 17 patients (8.02%).

**Table 1:** Baseline characteristics of the study population PCOM: Polycystic ovarian morphology

Baseline parameters	N=212, n (%)
Age (years) (mean±SD)	23.58 ± 4.6
Marital status	
Unmarried	111 (52.4­)
Married	101 (47.6)
Education	
Primary	1 (0.47)
Middle school	5 (2.36)
High school	7 (3.3)
Intermediate	62 (29.25)
Graduate	96 (45.28)
Postgraduate	41 (19.34)
Presenting complaints	
Irregular menstrual cycles	196 (92.5)
Hirsutism	92 (43.4)
Infertility	47 (22.2)
Acne	42 (19.8)
Heavy menstrual bleeding	10 (4.7)
Acanthosis nigricans	9 (4.3)
Ultrasound features of PCOM	
Yes	196 (92.5)
No	16 (7.6)

Anthropometric parameters

The average height of the study participants was 154.3 ± 5.33 cm, with an average weight of 61.2 ± 11.38 kg. The mean body mass index (BMI) was calculated at 25.8 ± 4.68 kg/m², while the average waist and hip circumferences were 81.6 ± 12.33 cm and 98.6 ± 8.65 cm, respectively. The waist-to-hip (W:H) ratio averaged 0.82 ± 0.08, with 58.5% of the participants having a W:H ratio greater than 0.8.

Hormonal parameters

The mean levels of key hormonal markers were as follows: LH at 8.52 ± 5.11 mIU/mL, FSH at 6 ± 1.87 mIU/mL, and the LH:FSH ratio at 1.49 ± 0.92. Additionally, the mean levels of TSH, serum prolactin, and total testosterone were 2.95 ± 1.51 µIU/mL, 12.66 ± 4.36 ng/mL, and 47.25 ± 19.53 ng/dL, respectively. Serum anti-Müllerian hormone (AMH) levels averaged 6.8 ± 3.56 ng/mL, with the majority (84.43%) of patients exhibiting levels above 4 ng/mL.

Metabolic parameters

The mean fasting insulin level among participants was 15.5 ± 8.52 µIU/mL, with 98.11% of patients recording fasting insulin levels below 37.6 µIU/mL. The mean fasting blood glucose level was 85.7 ± 10.98 mg/dL. The mean HOMA-IR value was 3.3 ± 2.01, with 58% of patients having a HOMA-IR value greater than 2.5. Regarding vitamin D levels, the average was 23.98 ± 13.8 ng/mL. Vitamin D deficiency was observed in the majority of patients (114 (53.8%)), followed by insufficient levels in 59 patients (27.8%), and sufficient levels in only 39 patients (18.4%). The detailed hormonal and metabolic parameters are presented in Table 2.

**Table 2:** Metabolic parameters of the study population ^a^mean±SD

Metabolic parameters	N=212, n (%)
Fasting Insulin (µIU/mL)^a^	15.5 ± 8.52
Fasting blood sugar (mg/dL)^a^	85.7 ± 10.98
HOMA-IR category^a^	3.3 ± 2.01
<2.5	89 (42.0)
≥2.5	123 (58.0)
Vitamin D (ng/mL)^a^	23.9± 13.8
Deficiency (<20 ng/mL)	114 (53.8)
Insufficient (20 to < 30 ng/mL)	59 (27.8)
Sufficient (30 to 100 ng/mL)	39 (18.4)

Correlation of anthropometric, hormonal, and metabolic parameters with PCOS phenotypes

Significant associations were found between certain clinical features and PCOS phenotypes. Acne prevalence was notably higher in Phenotype B compared to Phenotypes A, C, and D (p<0.001). Hirsutism was significantly more common in Phenotypes A, B, and C than in Phenotype D (p<0.001). Oligomenorrhea was more frequently observed in Phenotypes A, B, and D compared to Phenotype C (p<0.001). Acanthosis nigricans was significantly less common in Phenotype D compared to the other phenotypes (p=0.004).

Among the anthropometric measurements, only BMI showed a significant correlation with PCOS phenotypes (p=0.002), with higher BMI values in Phenotypes A and B compared to Phenotypes C and D. No significant associations were found between PCOS phenotypes and waist circumference (p=0.401), hip circumference (p=0.496), or W:H ratio (p=0.635).

For hormonal parameters, serum AMH levels were significantly associated with PCOS phenotypes, with the highest median AMH levels observed in Phenotype A and the lowest in Phenotype B. However, no significant associations were identified between the PCOS phenotypes and serum LH, FSH, LH:FSH ratio, TSH, prolactin, or total testosterone levels.

Regarding metabolic parameters, fasting insulin, fasting blood glucose, and HOMA-IR did not show significant correlations with PCOS phenotypes (Table 3).

**Table 3:** Comparison of baseline, anthropometric, hormonal, and metabolic parameters among PCOS phenotypes *mean±SD, ^†^n(%), ^‡^median (IQR), ^§^Kruskal Wallis test, ^||^Fisher's exact test, ^¶^ANOVA HOMA-IR: Homeostatic model assessment of insulin resistance

Parameters	Phenotype A (n=73)	Phenotype B (n=17)	Phenotype C (n=18)	Phenotype D (n=104)	p-value
Age (years)*	23.86±4.36	24.12±5.11	24.33±5.49	23.15±4.61	0.547^§^
Irregular menses^†^	71 (97.26)	16 (94.12)	10 (55.56)	104 (100)	<.0001^||^
BMI (kg/m^2^)^‡^	27.39±4.92	26.2±4.83	24.45±3.75	24.82±4.34	0.002^¶^
Waist-to-hip ratio^‡^	0.83(0.77-0.88)	0.82(0.78-0.88)	0.81(0.75-0.86)	0.81(0.76-0.85)	0.635^§^
LH (mIU/mL)^‡^	6.2(4.3-9.3)	8.02(6.5-10.6)	6.75(4.9-8.45)	8.2(4.97-12.47)	0.091^§^
FSH (mIU/mL)^‡^	5.8(4.9-7.04)	5.2(4.3-6.5)	5.25(4.73- 6.06)	5.91(5.03-7.5)	0.236^§^
LH:FSH ratio^‡^	1.1(0.73-1.6)	1.63(1.35-2.5)	1.31(0.87- 1.63)	1.39(0.97-1.96)	0.087^§^
TSH (μIU/mL)*	3.17±1.49	2.82±1.55	2.59±1.24	2.88±1.55	0.401^¶^
Serum prolactin (ng/mL)*	12.49±4.4	11.82±4.39	13.34±4.6	12.8±4.32	0.733^¶^
Total testosterone (ng/dL)*	49.57±21.15	47.33±23.31	43.14±17.59	46.33±18.03	0.563^¶^
Serum AMH (ng/mL)^‡^	9.34(5.8-11.56)	2.1(2.1-4.2)	4.22(3.73-5.08)	5.13(4.6- 6.89)	<.0001^§^
Fasting insulin (μIU/mL)*	16.12±9.05	12.63±5.09	12.61±5.23	16.03±8.91	0.186^¶^
Fasting blood sugar (mg/dL)*	87.79±11.39	84.24±9.27	84.33±9.24	84.62±11.13	0.239^¶^
HOMA-IR*	3.55±2.19	2.59±0.96	2.7±1.36	3.38±2.07	0.171^¶^
Vitamin D (ng/mL)^‡^	18(14.5-26)	19(17-23)	21.25(15.317-37)	19.56(16.3-29.55)	0.302^§^

**Figure 1:**
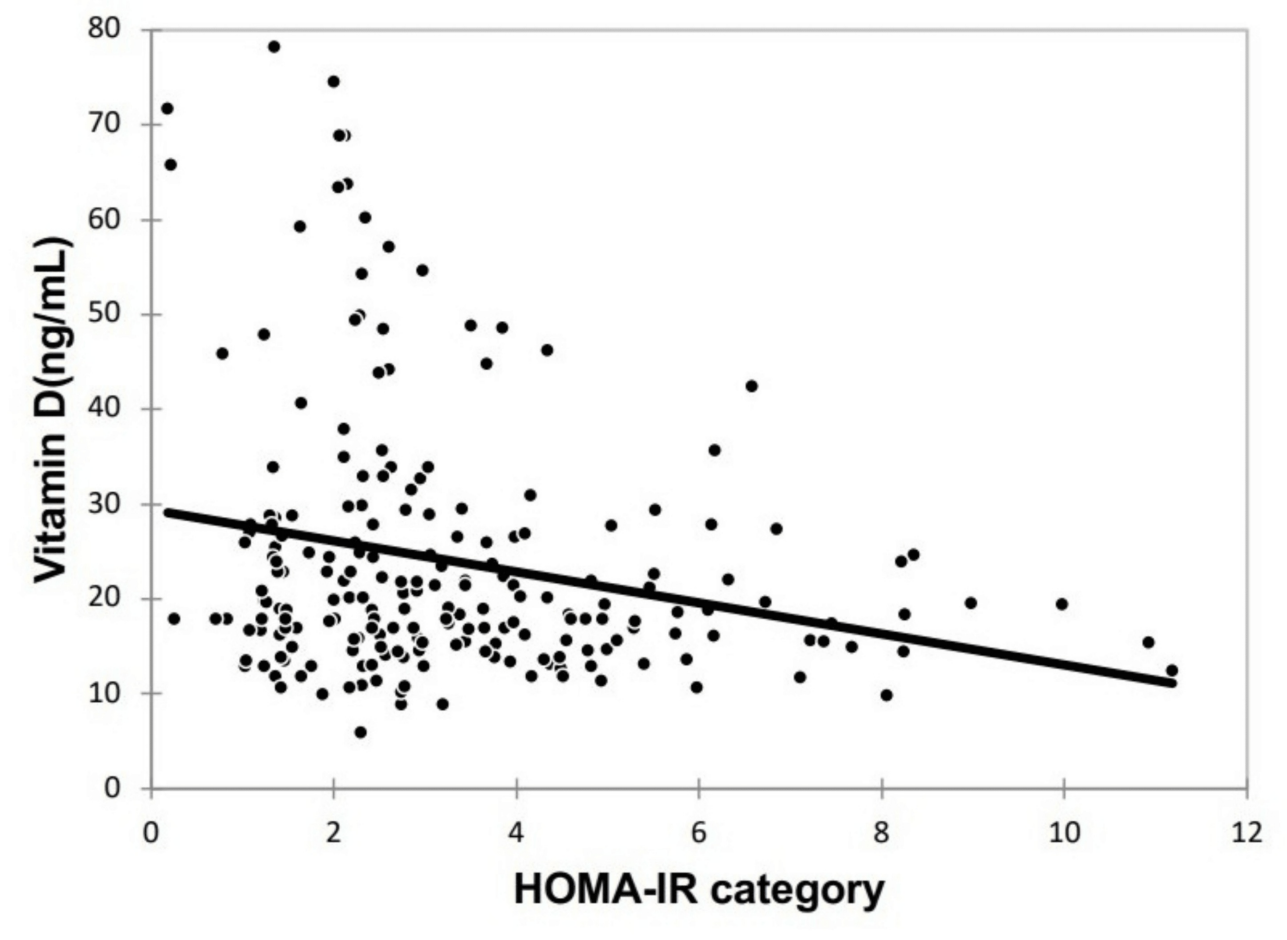
Correlation between HOMA-IR and vitamin D levels HOMA-IR: Homeostatic model assessment of insulin resistance

However, vitamin D deficiency was significantly more common among women with PCOS (p=0.029), although mean serum vitamin D levels did not vary significantly across the phenotypes. While the distribution of HOMA-IR categories was similar with respect to vitamin D levels (p=0.052), quantitative analysis revealed higher HOMA-IR values in the vitamin D-deficient (3.7 ± 2.25) and insufficient (3.09 ± 1.74) groups compared to the sufficient group (2.53 ± 1.28) (p=0.004). A significant negative correlation was found between HOMA-IR and vitamin D levels (correlation coefficient: -0.207), as depicted in Figure 1.

## Discussion

This prospective cross-sectional study evaluated various anthropometric, biochemical, and metabolic parameters in women with different PCOS phenotypes, revealing significant correlations between BMI, serum AMH levels, and PCOS phenotypes. Vitamin D deficiency was prevalent among the study participants, though it did not significantly correlate with specific PCOS phenotypes. However, a notable negative correlation was identified between vitamin D levels and HOMA-IR, suggesting that lower vitamin D levels might act as surrogate markers for insulin resistance in women with PCOS.

In this study, the highest mean BMI was observed in Phenotype A, followed by Phenotypes C, D, and B. This aligns with findings by Teede et al., who reported that women with PCOS tend to have higher BMI and longitudinal weight gain compared to women without the condition. They also found that each unit increase in BMI increased the risk of reporting PCOS by 9.2% [14]. Similarly, a systematic review and meta-analysis by Lim et al. (2012), which included 21 studies, found a 61% pooled prevalence of overweight or obesity in women with PCOS compared to controls [15].

The proportion of patients with serum AMH levels greater than 4 ng/mL was significantly higher in Phenotypes A and D compared to Phenotypes B and C. This finding is consistent with the study by Piouka et al. (2008), which reported a gradual decrease in AMH levels from Phenotypes A to D [16]. Likewise, Romualdi et al. (2016) observed higher serum AMH levels and larger ovaries in Phenotypes A and D compared to other groups [17]. Sahmay et al. (2018) further supported these findings, suggesting that serum AMH values correlate with PCO morphology, with the lowest values found in phenotypes lacking PCO morphology, such as Phenotype B [18].

Vitamin D deficiency was prevalent in this study, consistent with the findings of He et al., who reported widespread vitamin D deficiency in women with PCOS, with 25-hydroxy vitamin D (25OHD) levels ≤ 20 ng/mL in 67-85% of patients [19]. Several observational studies have linked reduced 25OHD levels to insulin resistance, menstrual and ovulatory disturbances, reduced fertility, hirsutism, hyperandrogenism, obesity, and increased cardiovascular risk [20-22]. Wang et al. (2020) also found significantly lower serum 25OHD concentrations in women with PCOS compared to controls, with higher prevalence rates of vitamin D deficiency and insufficiency among the PCOS group [23].

Our study further corroborates the significant negative correlation between HOMA-IR and vitamin D levels, as observed in studies by Wang et al. (2020), Mishra et al. (2016), Li et al. (2021), and Keshavarz et al. (2017) [23-26]. These findings suggest that vitamin D supplementation may potentially improve insulin resistance in women with PCOS.

PCOS is one of the most prevalent endocrine disorders affecting women of reproductive age, characterized by chronic anovulation and hyperandrogenism. The syndrome’s hallmark features include hyperandrogenism and polycystic ovary (PCO) morphology, with clinical manifestations such as menstrual irregularities, signs of androgen excess, and obesity. Insulin resistance, along with metabolic and hormonal disturbances, plays a central role in the development of various complications associated with PCOS. Classifying PCOS into phenotypes can aid in tailored treatment approaches, as clinical presentations vary across phenotypes. The prevalence of different phenotypes is influenced by demographic, ethnic, and lifestyle factors [1].

In our study, Phenotype D emerged as the most prevalent phenotype. A more detailed examination of the distribution of clinical and biochemical factors across phenotypes could be valuable for developing targeted interventions to improve patient outcomes and prevent complications associated with PCOS. Understanding these correlations will also enhance our comprehension of the disease’s pathogenesis.

The widespread vitamin D deficiency observed in PCOS women, particularly those with obesity and insulin resistance, underscores the importance of monitoring and potentially addressing this deficiency. The correlation between serum 25OHD levels and metabolic risk factors in PCOS suggests that vitamin D deficiency may exacerbate the condition. While there may be a role for vitamin D supplementation in managing PCOS, the current evidence is limited, and further randomized controlled trials are necessary to confirm the potential benefits of vitamin D supplementation in this population [8,27,28].

This study's strengths include its prospective design and the comprehensive evaluation of a wide range of anthropometric, biochemical, and metabolic parameters in a sizable cohort of women with PCOS. The classification of participants into different phenotypes allowed for a nuanced analysis of the correlations between these parameters and specific PCOS subtypes, providing valuable insights into the heterogeneity of the syndrome. However, the study has limitations, including its cross-sectional nature, which limits the ability to establish causal relationships. Serum testosterone measurement was performed using CLIA, which, although widely used, is less accurate than liquid chromatography-mass spectrometry, the current gold standard for androgen assessment. Additionally, the study was conducted at a single institution, which may affect the generalizability of the findings to broader populations.

## Conclusions

The present study highlights the significant association between PCOS phenotypes and key anthropometric and hormonal parameters, such as BMI and serum AMH levels. Additionally, the findings underscore the prevalence of vitamin D deficiency in women with PCOS, with a notable correlation between low vitamin D levels and increased insulin resistance. These insights can inform the development of predictive models for identifying metabolic abnormalities in PCOS patients. However, to further elucidate the pathogenesis of this multifaceted disorder, future research with larger, controlled cohorts that consider various demographic factors is essential. Such studies will enhance our understanding and potentially guide more targeted interventions for managing PCOS.
